# Developing single nucleotide polymorphism markers for the identification of pineapple (*Ananas comosus*) germplasm

**DOI:** 10.1038/hortres.2015.56

**Published:** 2015-11-25

**Authors:** Lin Zhou, Tracie Matsumoto, Hua-Wei Tan, Lyndel W Meinhardt, Sue Mischke, Boyi Wang, Dapeng Zhang

**Affiliations:** 1 Sustainable Perennial Crops Laboratory, USDA-ARS, Beltsville Agricultural Research Center, Beltsville, MD 20705, USA; 2 Daniel K. Inouye Pacific Basin Agricultural Research Center, USDA-ARS, Hilo, HI 96720, USA; 3 College of Horticulture, Nanjing Agricultural University, Nanjing 210095, Jiangsu, China; 4 Yunnan Forestry Technological College, Kunming 650224, Yunnan, China

## Abstract

Pineapple (*Ananas comosus* [L.] Merr.) is the third most important tropical fruit in the world after banana and mango. As a crop with vegetative propagation, genetic redundancy is a major challenge for efficient genebank management and in breeding. Using expressed sequence tag and nucleotide sequences from public databases, we developed 213 single nucleotide polymorphism (SNP) markers and validated 96 SNPs by genotyping the United States Department of Agriculture - Agricultural Research Service pineapple germplasm collection, maintained in Hilo, Hawaii. The validation resulted in designation of a set of 57 polymorphic SNP markers that revealed a high rate of duplicates in this pineapple collection. Twenty-four groups of duplicates were detected, encompassing 130 of the total 170 *A cosmos* accessions. The results show that somatic mutation has been the main source of intra-cultivar variations in pineapple. Multivariate clustering and a model-based population stratification suggest that the modern pineapple cultivars are comprised of progenies that are derived from different wild *Ananas* botanical varieties. Parentage analysis further revealed that both *A. comosus* var. *bracteatus* and *A. comosus* var. *ananassoides* are likely progenitors of pineapple cultivars. However, the traditional classification of cultivated pineapple into horticultural groups (e.g. ‘Cayenne’, ‘Spanish’, ‘Queen’) was not well supported by the present study. These SNP markers provide robust and universally comparable DNA fingerprints; thus, they can serve as an efficient genotyping tool to assist pineapple germplasm management, propagation of planting material, and pineapple cultivar protection. The high rate of genetic redundancy detected in this pineapple collection suggests the potential impact of applying this technology on other clonally propagated perennial crops.

## Introduction

Pineapple, *Ananas comosus* (L.) Merr., is a perennial herbaceous fruit crop belonging to the family Bromeliaceae. The crop is cultivated in all tropical and subtropical regions and ranks third in production among noncitrus tropical fruits, following banana (including plantain) and mango. The annual worldwide production reached 21.9 million metric tons in 2012 and the top seven producers (Brazil, Philippines, Thailand, Costa Rica, Indonesia, India, and China) jointly accounted for 90% of the global production (FAO, 2014).^1^ The pineapple plant is indigenous to South America.^2,3^ The putative center of origin is located in the Paraná–Paraguay River drainages between southern Brazil and Paraguay, based on the diversity distribution of related species and botanical varieties of pineapple in this region.^4–6^ However, the eastern part of the Guiana shield has also been hypothesized as the center of domestication for pineapple, based on the variation of chloroplast and nuclear DNA markers, the high level of phenotypic diversity, and the large number of primitive cultigens in this area.^7,8^ Pineapple was widely cultivated in tropical Americas before the arrival of Christopher Columbus, the first European to see this fruit, in 1493.^9^

The introduction of pineapple into Asia and the Pacific began with the Spaniards in the early sixteenth century and pineapple reached Africa in mid-sixteenth century.^4^ Since then, there have been multiple introductions and exchanges of germplasm among the pineapple producing countries. ^4^ Although many landraces and traditional cultivars exist in the Americas, only a few cultivars have been dispersed to Asia and Africa for use in commercial production.^4,10^ About 70% of the world’s production comes from a single cultivar, Smooth Cayenne,^10^ which is a highly productive pineapple excellent for canning.^11^ The current fresh fruit pineapple market is largely comprised of two cultivars bred by the Pineapple Research Institute, CO-2 and MD-2.^11^ Developing new cultivars with desirable resistance and postharvest traits will depend on the available germplasm of this species. The United States Department of Agriculture (USDA) - Agricultural Research Service pineapple germplasm collection in Hilo, Hawaii, is one of the major collections in the world, along with the collections maintained by EMBRAPA/CNPMF in Cruz das Almas, Brazil, and by CIRAD-FLHOR in Martinique. As part of the ARS National Clonal Germplasm Repository for Tropical and Subtropical Fruit and Nut Crops, the collection at Hilo currently maintains over 180 accessions of pineapple cultivars and their wild relatives.

As with many other tropical perennial crops, pineapple germplasm is almost exclusively maintained by vegetative propagation, by crowns, slips, suckers, or *in vitro* culture. Vegetative propagation has allowed the exchange of germplasm as clones among regions, countries, and continents. However, the exchange of vegetative planting materials has also resulted in problems for conservators of pineapple germplasm because records and labels of the cultivars have not always followed the same naming conventions, and accessions have limited information about their correct identity. Therefore, homonyms and synonyms are common among the names of pineapple cultivars and that restricts the sharing of information and materials among pineapple researchers and hampers the use of pineapple germplasm in breeding.^12–14^ Another major challenge for pineapple cultivar identification is that the protracted vegetative propagation has led to the accumulation of somatic mutations. Some mutations caused noticeable phenotypic effects and created intra-cultivar variation, which became the target of clonal selection.^10^ While these selected mutants are important in horticultural production, it is necessary to identify them so that breeders and genebank curators can efficiently conserve and use these genetic materials.

The utilization of biochemical and DNA molecular markers for pineapple germplasm management has been recently reviewed.^14^ Using isozyme markers, Aradhya *et al*. studied pineapple germplasm and found considerable variation within and between species of *Ananas*.^15^ In the Hawaii pineapple collection, they identified 66 distinct zymotypes that were able to differentiate all species and botanical varieties. Their results also suggested that, rather than genetic divergence due to reproductive isolating barriers, differentiation among the species of *Ananas* may be due to ecological isolation, and therefore may represent a species complex.

Both dominant DNA markers (amplified fragment length polymorphism, AFLP) and co-dominant markers (restriction fragment length Polymorphism simple sequence repeat, SSR) have been used to assist pineapple cultivar identification and germplasm management.^16–23^ In spite of the significant progress in marker-assisted germplasm management over the last 20 years, cultivar identification in pineapple remains a challenging task. Using AFLP markers, Kato *et al.* characterized 148 *A. comosus* accessions maintained in the USDA pineapple collection in Hilo, Hawaii.^20^ They showed that a unique profile for major groups that had been classified by morphological traits, such as ‘Cayenne’, ‘Spanish’, and ‘Queen’, could not be established using AFLP-based DNA fingerprints. SSR markers likewise lacked congruence between phenotype and molecular marker-based classification in pineapple.^22,23^ Moreover, neither AFLP nor SSR are the most suitable marker tool for detection of duplicates in the pineapple germplasm.

Single nucleotide polymorphisms (SNPs) are the most abundant class of polymorphisms in plant genomes. Compared to SSR markers, SNP analysis can be done without requiring DNA separation by size and can, therefore, be automated in high-throughput assay formats. The diallelic nature of SNPs facilitates a much lower error rate in allele calling and promotes compatibility between laboratories. These advantages have resulted in the increasing use of SNPs as the markers of choice for accurate genotype identification and diversity analysis in perennial crops, as recently demonstrated in cacao (*Theobroma cacao*, 2013),^24^ grapevine (*Vitis vinifera*, 2011),^25^ pummelo (*Citrus maxima*, 2014),^26^ strawberry (*Fragaria* spp, 2013),^27^ tea (*Camellia sinensis*, 2014), and longan (*Dimocarpus longan*, 2015). Like other perennial horticulture crops, DNA fingerprinting that uses a small set of SNP markers is in great demand by the pineapple community for a broad range of research and field applications. These applications include, but are not limited to, identification of mislabeled accessions, parentage, and sibship analysis for quality control in breeding and seeds programs, and authentication and traceability to support the production of high-value clones for premium markets. Nonetheless, this most powerful tool for germplasm management has not been applied to pineapple germplasm management.

Ample genomic resources have been developed for pineapple.^14,28–31^ The premier online database, ‘PineappleDB’ (http://genet.imb.uq.edu.au/Pineapple/index.html), includes a more than 5,600 expressed sequence tags (ESTs) with 3,383 consensus sequences. The comprehensive sequence, bioinformatics, and functional classification of EST resources are available for text or sequence-based searches. A draft genome of *A. comosus* has been developed, which covers about 375 Mb (62%) of the estimated 526 Mb genome of this species.^14^ These readily available genomic resources provide opportunities for mining new markers to use for pineapple germplasm management and breeding. The objectives of the present study were to develop SNP markers through the data mining of ESTs and transcriptome data and to assess their potential application for pineapple cultivar identification. The results reported herein represent the first validation study of SNPs in pineapple, demonstrating the utility of a transcriptome as an approach for rapid development of a high-quality genotyping tool. These SNP markers, as well as the genotyping method, will be particularly useful for intellectual property rights in varietal protection, germplasm management, and pineapple breeding programs.

## Materials and methods

### Mining of putative SNPs from EST and nucleotide sequences

All available nucleotide sequences of *Ananas* spp. were downloaded from NCBI GenBank (http://www.ncbi.nlm.nih.gov, 4 October 2014). Redundant entries were examined and excluded using the CD-HIT program with a 95% sequence similarity threshold. The FASTA-formatted files of pineapple were merged into a single data set for further data mining. Putative EST-SNPs were detected using the QualitySNP program.^32^ All of these selected clusters included a minimum of six EST sequences, whereas both the minimum redundancy threshold and minimal confidence score required by QualitySNP was set at three. In order to meet the requirements and constraints for primer design, all candidates for SNP markers with less than 50 nucleotides between two neighboring SNPs were removed. A subset of 96 identified SNP sequences was then chosen for design and manufacture of SNP assay.

### Validation of putative SNPs

To evaluate the putative SNP markers for suitability of cultivar identification, we used a nanofluidic genotyping system and validated the SNPs for 170 pineapple accessions (Table 1; Supplementary Table S1). The pineapple germplasm samples were from the pineapple collection maintained by the USDA-ARS Tropical Plant Genetic Resources and Disease Unit, at the National Plant Germplasm Repository in Hilo, Hawaii (http://www.ars.usda.gov/main/site_main.htm?modecode=20-40-05-10) were harvested and dried in silica gel. DNA was extracted from dried pineapple leaves with the DNeasy Plant Mini kit (Qiagen Inc., Valencia, CA, USA), which is based on the use of silica as an affinity matrix. The dry leaf tissue was placed in a 2-mL microcentrifuge tube with one quarter-inch ceramic sphere and 0.15 g garnet matrix (Lysing Matrix A; MP Biomedicals. Solon, OH, USA). The leaf samples were disrupted by high-speed shaking in a TissueLyser II (Qiagen Inc.) at 30 Hz for 1 min. Lysis solution (DNeasy kit buffer AP1 containing 25 mg/mL polyvinylpolypyrrolidone), along with RNase A, was added to the powdered leaf samples and the mixture was incubated at 65 °C, as specified in the kit instructions. The remainder of the extraction method followed the manufacturer’s suggestions. DNA was eluted from the silica column with two washes of 50 µL buffer AE, which were pooled, resulting in 100 µL DNA solution. Using a NanoDrop spectrophotometer (Thermo Scientific, Wilmington, DE, USA), DNA concentration was determined by absorbance at 260 nm. DNA purity was estimated by the 260:280 ratio and the 260:230 ratio.

Ninety-six putative SNP sequences were submitted to the Assay Design Group at Fluidigm Corp. (South San Francisco, CA, USA) for design and manufacture of primers for a SNPtype genotyping panel. The assays were based on competitive allele-specific PCR, and they enable bi-allelic scoring of SNPs at specific loci (KBioscience Ltd, Hoddesdon, UK). The Fluidigm SNPtype Genotyping Reagent Kit was used according to the manufacturer’s instructions.^34,35^ Using these primers, the isolated DNAs were subjected to Specific Target Amplification in order to enrich the SNP sequences of interest.^34^ Genotyping was performed on a nanofluidic 96.96 Dynamic Array IFC (Integrated Fluidic Circuit; Fluidigm Corp.). This chip automatically assembles PCRs, enabling simultaneous testing of up to 96 samples with 96 SNP markers. The use of a 96.96 Dynamic Array IFC for SNP genotyping of human samples has been described by Wang *et al*.^33^ End-point fluorescent images of the 96.96 IFC were acquired on an EP1 imager (Fluidigm Corp.). The data were analyzed with Fluidigm Genotyping Analysis Software (Fluidigm Corp.).^36^

### Data analysis

Duplicate accessions were identified using pairwise multilocus matching among all individual samples. DNA samples that were fully matched at the genotyped SNP loci were declared the same cultivar or clones. The program GenAlEx 6.5 (2006, 2012) was used for computation.^37,38^

After duplicate identification, the redundant samples were removed and descriptive statistics for measuring the informativeness of the SNP markers were calculated based on the remaining distinctive cultivars. The key descriptive statistics included minor allele frequency, observed heterozygosity, expected heterozygosity, Shannon’s information index, and inbreeding coefficient. Computations were carried out using the same program.

A cluster analysis using the neighbor-joining (NJ) method was used to further examine the genetic relationship among accessions. Kinship coefficient was chosen as genetic distance measurement of shared ancestry among the individual accessions. The computation was executed using MICROSATELLITE ANALYZER (MSA, 2003).^39^ A dendrogram was generated from the resulting distance matrix using the NJ algorithm available in PHYLIP.^40,41^ The unrooted tree was visualized using the web-based tool Interactive Tree of Life v2 (http://itol.embl.de/).^42^

A model-based clustering algorithm implemented in the STRUCTURE software program was applied to the SNP data.^43^ This algorithm attempted to identify genetically distinct subpopulations based on allele frequencies. The admixture model was applied and the number of clusters (K-value), indicating the number of subpopulations the program attempted to find, was set from 1 to 10. The analyses were carried out without assuming any prior information about the genetic group or geographic origin of the samples. Ten independent runs were assessed for each fixed number of clusters (K), each consisting of 1 × 10^6^ iterations after a burn-in of 2 × 10^6^ iterations. The ΔK value was used to detect the most probable number of clusters and the computation was performed using the online program STRUCTURE HARVESTER.^44^ Of the 10 independent runs, the one with the highest Ln Pr (X|K) value (log probability or log likelihood) was chosen and represented as bar plots.

To test the hypothesis that vars. *ananassoides*, *bracteatus*, and *erectifolius* are the putative progenitors for cultivated pineapples, we applied parentage analysis to verify the origin of the 53 accessions in *A. comosus* var. *comosus* (as labeled in Table 1). These cultivars or breeding lines were considered as ‘offspring’ for which parentage analyses were carried out. *A. comosus* vars. *ananassoides*, *bracteatus*, and *erectifolius* were used as candidate parents. A likelihood-based method implemented in the program CERVUS 3.0 was used for computation.^45,46^ For each parent–offspring pair, the natural logarithm of the likelihood ratio (LOD score) was calculated. Critical LOD scores were determined for the assignment of parentage to a group of individuals without knowing the maternity or paternity. Simulations were run for 10 000 cycles, assuming that 10% of candidate parents were sampled, a total of 90% of loci was typed with a 1% typing error rate. The most probable single mother (or father) for each offspring was identified on the basis of the critical difference in LOD scores (D) between the most likely and the next most likely candidate parent at greater than 95 or 80% confidence.^45,46^

## Results

### SNP discovery

A total of 13 203 mRNA nucleotide and 5941 EST sequences from pineapple were gathered using methods previously described. After adapter removal, trimming, and quality control, 18 241 higher quality sequences were selected. The program CAP3,^47^ using default parameters, was used to assemble sequences into 1793 contigs and 11 809 singlets with an average size of 3.59 sequences per contig, among which putative SNPs were detected in only 48 contigs using the QualitySNP program. Each of these selected clusters included a minimum of six EST sequences. In total, we obtained 213 putative SNPs, including 75C/T, 59A/G, 10A/T, 12A/C, 4T/G, 11C/G, 41 indel, and 1 high tri-allelic polymorphism. To select high-quality SNPs for validation, candidate SNP sites with at least 50 bp before and after the site were filtered. We calculated the number of all sequences in a cluster and the number containing the SNP type in this cluster. We then selected 96 SNPs for validation by genotyping the 170 pineapple accessions in the USDA-ARS pineapple collection.

### Screening for polymorphic SNP markers

Out of the chosen 96 SNP markers, 80 were successful for genotyping. The failure of the remaining 16 SNPs was likely due to the sequence complexity or the presence of polymorphisms within the flanking sequences. However, among the 80 successful SNPs, 23 were monomorphic across the 170 pineapple samples (i.e. only one SNP variant was identified in all individuals). These monomorphic markers may have resulted from errors in transcriptome sequencing, which then led to the incorrect identification of SNP. It is also possible that some of these SNPs may correspond to rare alleles that were not present in the set of pineapple accessions we analyzed. A total of 57 polymorphic SNPs were retained for further analysis of this sample set. These 57 SNPs were reliably scored across the validation panel and thus were considered true SNPs. The flanking sequences of these 57 SNPs are listed in Table 2.

### Cultivar identification

**Figure 1:**
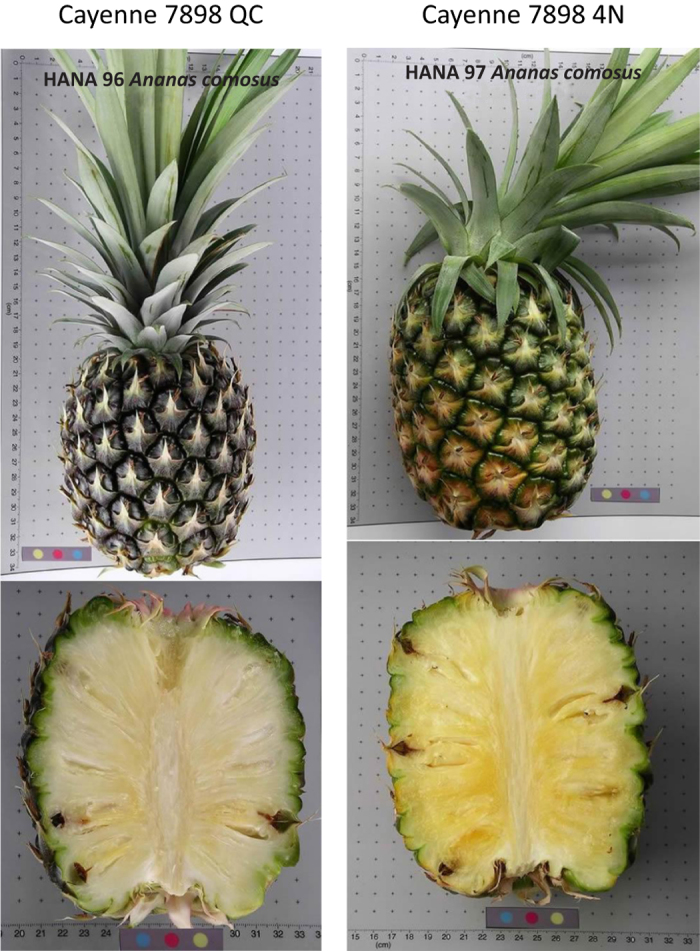
Somaclonal mutation of clone ‘Cayenne 7898’ showing the difference in dark yellow (Cayenne 7898 4N, HANA 97) and white (Cayenne 7898, HANA96) flesh colors.

SNP profiles of the multiple accessions from the same pineapple cultivar showed that genotyping results were highly consistent (Table 3). Multilocus matching of SNP fingerprints revealed a high rate of duplicates in this pineapple collection. A total of 130 accessions could be classified into 24 synonymous groups (Table 4). The largest synonymous group, which includes 36 accessions, was found in cultivar Cayenne. It is also noticeable that some accessions within the same synonymous group have apparent morphological differences, despite matching SNP profiles, indicting somaclonal mutation within the synonymous group. For example, Cayenne 7898 QC has atypical yellow flesh color, whereas Cayenne 7898 4N has a white color, but their SNP profiles are the same (Figure 1).

### Descriptive statistics and clustering analysis of 64 distinctive pineapple accessions

From each of the synonymous groups, only one accession was retained and used for subsequent diversity analysis. Among the 170 genotyped accessions, there were 64 accessions with a unique SNP profile. Descriptive statistics were then computed for the 57 polymorphic SNPs across the 64 pineapple accessions with a unique SNP profile and the result is presented in Table 5. The minor allele frequencies of these 57 SNPs ranged from 0.090 to 0.495 with an average of 0.324. The mean information index was 0.601, ranging from 0.304 to 0.693. The observed heterozygosity ranged from 0.110 to 0.935 with an average of 0.520, whereas the mean expected heterozygosity was 0.414 ranging from 0.164 to 0.500 (Table 5).

**Figure 2:**
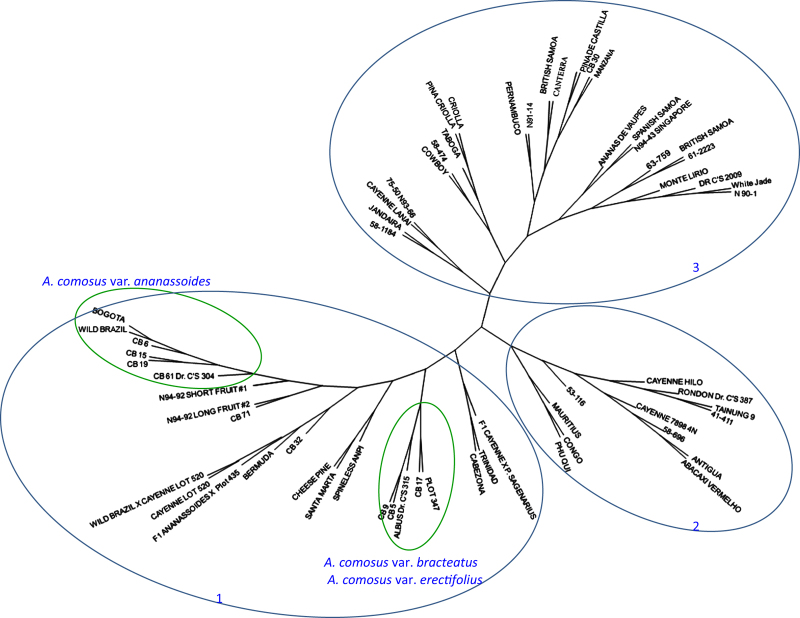
NJ unrooted tree depicting the relationship among 64 pineapple accessions from USDA-ARS, Pacific Basin Tropical Plant Germplasm Resource Center in Hilo, Hawaii. Identification of accessions corresponds to samples listed in Table 1 and Supplemental Table 1.

The unrooted NJ tree grouped the 64 accessions into three main clusters (Figure 2). The clustering patterns presented relationships among accessions based on the different botanical varieties or origins from different geographical regions. The first cluster includes all the accessions of *A. comosus* vars. *ananassoides*, *bracteatus*, and *erectifolius,* as well as the hybrids derived from these related botanical varieties. Within this cluster, vars. *ananassoides bracteatus* and *erectifolius* are clearly separated. This cluster also included several cultivated pineapple clones, such as Bogota, Pina Lisa, and Criolla from Colombia, Bermuda from Barbados, Cayenne Lot 520 from Hawaii, Cabezona from Puerto Rico, and Trinidad from Trinidad. The proximity between these cultivars and the two related botanical varieties indicates that these cultivars are either selected or derived from vars. *ananassoides* and *bracteatus.* The two Bolivian accessions (N94-92 Short Fruit#1 and N94-92 Long Fruit#2) were labeled as *Ananas* species in their passport record data. The cluster result showed that they should be *A. comosus* var. *ananassoides* or hybrids derived from *A. comosus* var. *ananassoides*.

The second cluster comprised of exclusively *A. comosus* var.*comosus*, including several well-known cultivars such as Cayenne Hilo, Mauritius, and Antigua. Since these three cultivars represent the reference horticultural groups of ‘Cayenne’ (Cayenne Hilo) and ‘Queen’ (Mauritius and Antigua), respectively, their grouping here, in one main cluster, demonstrated that the differences used to designate membership to these two horticultural groups are relatively small, in comparison with the other botanical varieties. The third cluster includes 26 cultivated pineapples that formed a large and diverse group. Within this large cluster there are several important pineapple cultivars such as Criolla from Mexico, Montelirio from Guatemala, and Pernambuco and Manzana from Brazil. The majority of the accessions in this cluster seemed mainly cultivated in South America.

### Assignment test by STRUCTURE

**Figure 3:**
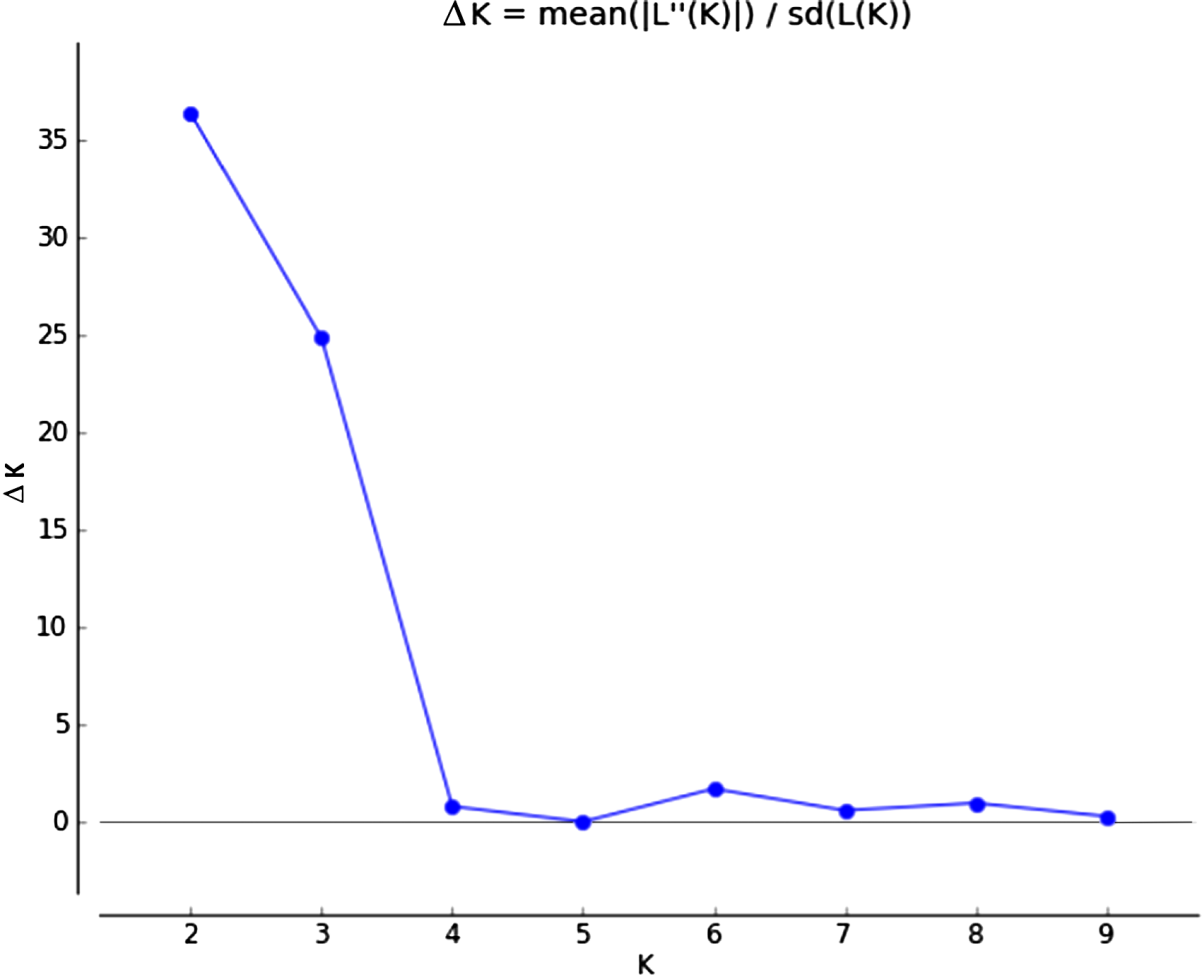
Plot of ΔK (filled circles, solid line) calculated as the mean of the second-order rate of change in likelihood of K divided by the standard deviation of the likelihood of K, m|L″(K)|/s[L(K)].

**Figure 4:**
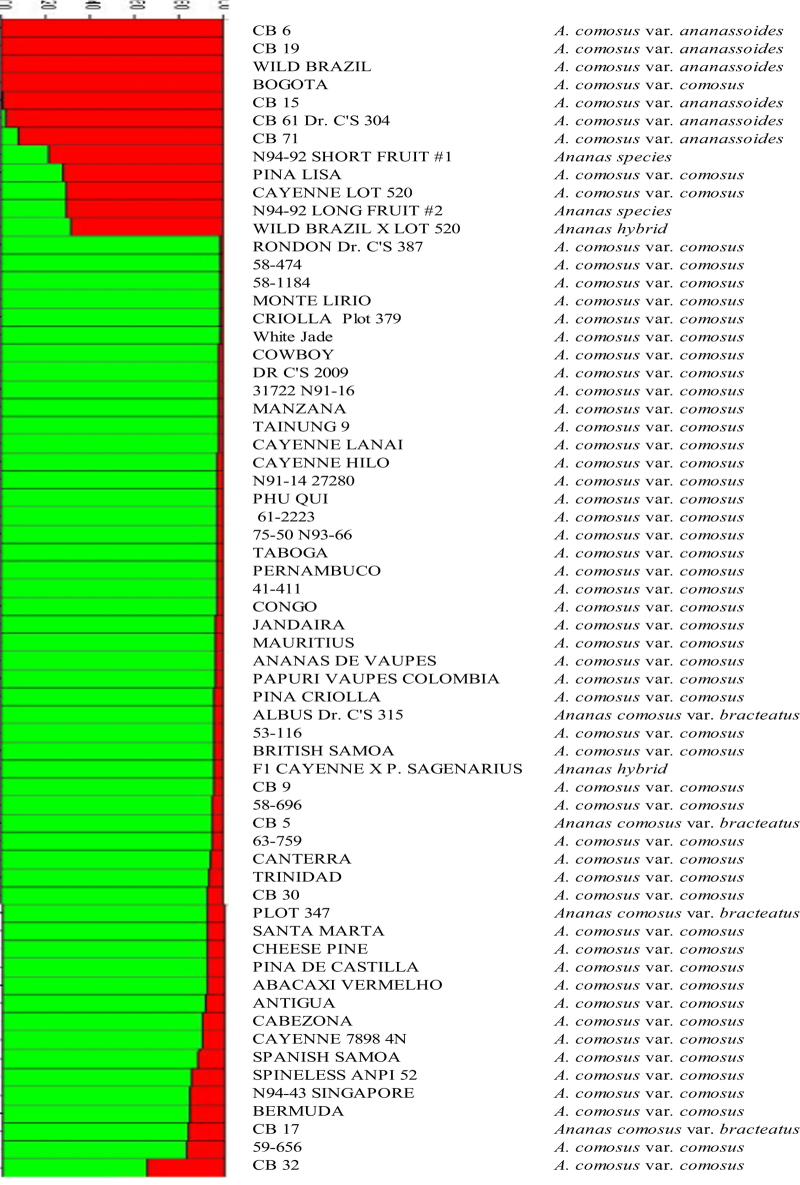
Inferred clusters in the pineapple accessions varieties using STRUCTURE in the overall analyzed pineapple accessions. Each vertical line represents one individual multilocus genotype. Individuals with multiple colors have admixed genotypes from multiple clusters. Each color represents the most likely ancestry of the cluster from which the genotype or partial genotype was derived. Clusters of individuals are represented by colors.

Population stratification of the 64 accessions, based on ΔK value computed by STRUCTURE HARVESTER, revealed two clusters as the most probable number of K (Figures 3 and 4) and this partitioning was largely compatible with the cluster analysis (Figure 2). All the accessions related to var. *ananassoides* were assigned to one Bayesian cluster, whereas the cultivated germplasm, as well as vars. *bracteatus* and *erectifolius*, were grouped in a different cluster. The F1 hybrid of Wild Brazil × Plot 520 was confirmed by analysis with STRUCTURE. In addition, several accessions were classified as hybrids of the two clusters, such as N94-92, F1 Ananassoides × Plot 435, Wild Brazil × Cayenne Lot 520, and Cb 32 (Figures 3 and 4). The result of assignment by STRUCTURE is largely compatible with the result of clustering analysis (Figure 4). All the accessions assigned by STRUCTURE in the cluster of var. *ananassoides* or its hybrids were in the first cluster of the NJ tree.

### Parentage analysis

Among the 52 cultivars and hybrids derived from related botanical varieties, paternal or maternal parents were assigned (>80% confidence level) to 14 accessions (Table 6). *A. comosus* var. *ananassoides* was responsible for parentage of three accessions including Bogota, Bermuda, and Pina Lisa, whereas *A. comosus* var. *bracteatus* was assigned to parentage of 10 accessions. No parentage was assigned to *A. comosus* var. *erectifolius*. The result of parent–offspring assignment is largely compatible with the cluster analysis (Figure 2). Accessions assigned as offspring from the same parent tended to be grouped together in the NJ tree (Figure 2). For example, CB 17 was found to be the likely progenitor for Mauritius, Phu Qui, and Congo, all of which grouped together in the same subcluster in group 3 (Figure 2).

## Discussion

Despite substantial progress in genomics research on pineapple, advanced molecular tools to support germplasm management are not available. Developing SNP markers from transcriptome sequences has been considered an efficient strategy for non-model species. In the present study, we validated 96 SNP markers based on the transcriptome sequences of pineapple at various development stages and used them to genotype a diverse panel of cultivated and wild germplasm. We obtained a success rate of approximately 60% for marker validation, which demonstrated that this approach can serve as a shortcut for SNP development. As shown in the present study, even a small set of SNP markers can significantly improve accuracy and efficiency in germplasm management.

### Pineapple cultivar identification

Reliable identification of pineapple cultivars is invaluable for germplasm conservation and cultivar protection. In the present study, it has been demonstrated that the set of 57 SNP markers was effective for the assessment of genetic identity of pineapple germplasm. Results from multiple clones of the same cultivar showed 100% concordance, demonstrating that the nanofluidic system is a reliable platform for generating pineapple DNA fingerprints with high accuracy. The present result revealed a high rate of genetic redundancy in this pineapple collection. Some of the identified duplicates are well-documented synonymous cultivars. For example, the Cayenne cultivars are known to be derived from a few ancestral pineapple plants that originated from Cayenne, French Guiana.^10,48^ But majority of the clones or synonymous groups have been less known to the pineapple community, such as Pernambuco vs. Sugar Loaf, Spanish Samoa vs. Natal, and Ruby vs. Los Banos. Identification of these clone groups will significantly facilitate the efficient exchange, conservation, and use of pineapple germplasm.

However, caution is needed for the interpretation of genetic redundancy in pineapple. It is well known that somatic mutation is common in pineapple. Many phenotypic traits such as spiny leaves, fruit flesh color, acidity, and sugar content of fruit have been well documented. These somatic mutations are the major source of variation exploited for the selection of new cultivars. For example, the spiny or smooth leaf margins, caused by a single gene,^10^ are the signature character for the cultivar group Smooth Cayenne. Such a mutation is difficult to detect when a small set of molecular markers are applied. Similar problems were found in fingerprinting projects dealing with other vegetative propagated crops such as bananas (*Musa* spp., 2014), ^49^ bread fruit (2015),^50^ and apple (*Malus* spp., 2012).^51^ More comprehensive genomic approaches, such as next-generation sequencing, would be needed to detect which genes or alleles had been changed, thereby causing the phenotypic variation. For this reason, the reduction of identified duplicates in pineapple germplasm genebank needs to be considered on a case-by-case basis. Characterization of phenotypic traits among the synonymous group members is still essential to complement DNA fingerprinting for genotype identification.

### Classification of pineapple germplasm

*A. comosus* is a mostly self-incompatible diploid with 2n=2x=50 chromosomes.^52,53^ This species includes five botanical varieties of *A. comosus*: vars. *comosus*, *ananassoides*, *parguazensis*, *erectifolius*, and *bracteatus*, based on the revised classification of Coppens d’Eeckenbrugge and Leal.^7^ The present results show that out of the 170 *Ananas* accessions maintained in the USDA pineapple collection, there are only 64 distinctive genotypes. Clustering analysis and model-based stratification both showed that *A. comosus* var. *ananassoides* differs from *A. comosus* var. *bracteatus*, thus supporting the revised taxonomy system that classified *A. comosus* var. *ananassoides* and var. *bracteatus* as two different botanical varieties.^7^ However, accessions of var. *erectifolius* were found to have high similarity and grouping closely together with the accessions of var. *bracteatus*. This result differs with a previous report based on isozyme variation,^15^ which showed that *A. comosus* var. *erectifolius* did not have a distinctive isozyme profile, in comparison with the rest of the *A. comosus* var. *comosus* cultivars. Nonetheless, the present study only used two accessions of *A. comosus* var. *erectifolius*, which may be a bias in terms of the sample representation. Additional samples of *A. comosus* var. *erectifolius* from other genebanks need to be examined and a larger number of SNP markers need to be analyzed to clarify if the classification of *A. comosus* var. *erectifolius* should be revised.

The second observation is that several cultivated pineapple accessions (Bogota, Pina Lisa, Bermuda, Cayenne Lot 520, Cabezona, and Trinidad) were grouped together with *A. comosus* var. *ananassoides* or *A. comosus* var. *bracteatus*, instead of with the rest of the *A. comosus* var. *comosus* accessions (Figures 2 and 3). This result indicates that the current system that classifies all cultivated pineapple into a single botanical variety (*A. comosus* var. *comosus)* may be questionable. It would be appropriate to consider cultivated pineapple as a complex of different botanical varieties, with possible significant gene flow among them.

The third observation is about the validity of the horticultural classification of pineapple germplasm. Pineapple cultivars are classified into several horticultural groups. The commonly known groups include ‘Abacaxi’, ‘Cayenne’, ‘Maipure’, or ‘Perolera’, ‘Queen’, and ‘Spanish’.^10,54,55^ Despite these horticultural groups having been adopted by many users, little investigation has been done to show a genetic basis to reinforce this categorization. Kato *et al.* examined the efficacy of the horticultural groups and reported that the classifications of ‘Cayenne’, ‘Spanish’ and ‘Queen’ were not well supported by AFLP analysis.^20^ Shoda *et al.* analyzed 31 pineapple accessions using SSR markers.^22^ Their results also showed disagreement between the horticultural type and the results of the SSR analysis. The current study showed that the ‘Cayenne’ cultivars have a distinguishable genetic identity, and most of the affiliated accessions were grouped in a single cluster. However, accessions in the other groups did not appear well clustered. For example, cultivars Mauritius and Antigua are two well-known reference cultivars in the ‘Queen’ group, but in the NJ tree (Figure 2) they were separated in different subclusters, where cv. Antigua showed higher proximity with the ‘Cayenne’ group than with Mauritius. Similar discordance was found between cultivars of the ‘Spanish’ group (Figure 2). Therefore, our results support the previous conclusions of Kato *et al*.^20^ and Shoda *et al*.^22^ that the classification of pineapple cultivars into horticultural groups lacks consistency in terms of their genetic bases. Revision seems needed on this classification with the support of new evidence generated by DNA markers.

### Putative progenitors of pineapple

Parentage analysis showed that both vars. *bracteatus* and *ananassoides* can be progenitors of pineapple cultivars (Table 6). This result is in agreement with the fact that both var. *bracteatus* and var. *ananassoides* can intercross successfully with var. *comosus* to produce fertile offspring.^7,56^ Coppens d’Eeckenbrugge and Leal hypothesized that var. *ananassoides* is the likely progenitor of cultivated pineapple, and it is likely that domestication happened in the Guiana shield.^7^ One strong piece of evidence supporting this hypothesis is that all four chloroplast haplotypes that have been identified in cultivated materials are present in the wild var. *ananassoides*.^7^ On the other hand, var. *bracteatus* was not considered as a progenitor in this hypothesis, mainly because var. *bracteatus* appeared to be a homogeneous variety with narrow genetic diversity, which is an unlikely basis for diverse domesticated cultigens of pineapple.^7^ The present result, however, shows that 11 pineapple cultivars (Canterra, Papuri Vaupes Colombia, CB 30, Pina de Castilla, Rondon, Congo, Phu Qui, Mauritius, Cheese pine), which are dispersed across different clusters as shown in the NJ tree, Figure 2), could have their parentage (either male or female) traced back to var. *bracteatus* (Table 6). *Ananas comosus* var. *bracteatus* is native to Brazil, Bolivia, Argentina, Paraguay, and Ecuador but not to the Guiana shield. The present result thus indicates the possibility that pineapple could have been domesticated at multiple sites, involving both var. *ananassoides* and var. *bracteatus.* The Parana-Paraguay river drainage area could be one of the domestication sites, since both var. *bracteatus* and var. *ananassoides* are indigenously distributed in this area.^4,5^ Geographically disparate origins of crop domestication are not uncommon in the Americas, as in the case of common bean (*Phaseolus vulgaris*), chili pepper (*Capsicum* spp.), potato (*Solanum* spp.), and cacao (*T. cacao*), as reviewed by Clement *et al*.^57^

In conclusion, we conducted a study to develop a set of SNP markers for pineapple and employed them for fingerprinting the USDA’s pineapple collection, using a nanofluidic array. This approach enabled us to generate high-quality SNP profiles for the purpose of pineapple cultivar identification. This is a highly useful tool for genebank management, which will also lead to more efficient crop improvement and, furthermore, has the potential to protect intellectual property rights of breeders. Our result also generated significant insight regarding the origin and domestication of pineapple. Efforts to sequence multiple cultivars from the same synonymous groups with somaclonal mutations are underway, in order to gain a comprehensive understanding about the genetic basis for mutation-based changes in important agronomic traits. This information will be highly useful for verification of pineapple cultivars and will improve the efficiency of pineapple genebank operation. The high rate of genetic redundancy detected in this collection, also suggests the potential impact of applying this technology on other tropical perennial crops.

## Figures and Tables

**Table 1:** Species categories of 170 *A. comosus* accessions in USDA-ARS pineapple collection at Hilo, Hawaii.

Species	No. of accessions
*A*. *comosus* var. *comosus*	145
*A*. *comosus* var. *ananassoides*	6
*A*. *comosus* var. *bracteatus*	8
*A*. *comosus* var. *erectifolius*	2
*A*. *comosus* hybrid	5
*Ananas* species	4
Total	170

**Table 2:** Flanking sequences and SNPs of the 57 polymorphic markers.

SNP ID	Flanking sequences and SNPs
Ac4	TAAGCGACGCCGGTGAATTGACCTCATTGATGGACTCCGACAAATATGC[A/G]AAGTTCTGCGAGGAAGAAAATGCAAAACATTGAATGCATTTGCGTTGGCTC
Ac5	AAGCGAGCAAAGCTCTGTCCGTAGATCTCTCGGATTCTTAAGAGCCAAG[A/T]GTTCATGTCTGATCTCGACATCCAGATCCCAACTACCTTCGATCCGTTTGC
Ac6	CAAAATTCATGGTTTCTGAGCGGAGCTGCTGCTGACTACAAAATTGCAG[T/G]GGCCGGAAGTGTTAAGTTCAACTACAACTACTAAGCTTACGATCACTACGC
Ac9	CAAAAAATTGGGAGGAAAAATGATGAGTTGTTAAATAGACAGGTGCCAC[G/A]AAGAATGTAATCCTATATGTAACTGCAGATCTTTTGGAGCTGGAATGTGG
Ac10	ATATCTACGGTGATGTTATTAACAAAGCCAAATTTGCTATTAACAGAGA[G/A]ATTCACTGAATTCGCAGAAGTTTAGTACATTTGAACGATTCAATGAATCTA
Ac11	AGCATCCTGGAGGTGATGAGGTCTTGCTAGCTGCAACTGGGAAAGATGC[T/G]ACCAATGATTTTGAAGATGTGGGCCACAGCAACTCTGCGAGGGAAATGATG
Ac13	AACAAGCATCATACAATCCCGACAAGTCTTCGGATTTCCTTATCAAAAT[G/A]CTGCAGTTTCTTGTGCCTATCTTGATCTTGGGCTTGGCATTTGCCGTCCGG
Ac15	GGACTCGGGACTCGTTCCCGGAGGTTCGCGCTTCTCGTCGACGACCTCA[C/A]GGTGAAGGTCGCAAACATCGAGGAGGGCGGCCAGTTCACCATATCGGGGGC
Ac17	GCGGAGAACCAATCGCTCTGGATGTCCCTGCTAGACACATCTTTTCACG[A/G]TCCAGGATTCGCCACAAGGACAACATCAACGCTTTCTTGGATTCTAATGGT
Ac19	CTTCAGTGTGTGGCCGCTACACAGTGTTGTGCACACGCACACGCGCACA[T/C]GCTTTTATTTATTTGTTCTGTATAGTTCGTTCAGTGTGTTGTAGGTATAGA
Ac20	CGACTGGATGTACAGTGGAGAAACAAGGAGCAACCACCGTTGAGATCGG[G/A]GAAATGGAGGAGTGGAGCACTTGGTGGTGGTGGTGGAGGTGGTGTTGGGAG
Ac21	GCACTTGGTGGTGGTGGTGGAGGTGGTGTTGGGAGGAGTAGTAAATGGG[T/C]TGTGTAAATTTTGTTCCTCTGCTGCATGTTTGCTGTTTTATGTGTGCAAAT
Ac31	ATATACACTTTGACAATTACGCCTGGTTGGGAGGGAGTCTGTGCCATGG[C/T]GAGAACAATTTGCAGCTGGGTTTCCCTACTCCCATTTGCCCAACAATAATC
Ac32	GGTTGGGAGGGAGTCTGTGCCATGGCGAGAACAATTTGCAGCTGGGTTT[C/T]CCTACTCCCATTTGCCCAACAATAATCACCATCCTTCAAAGCCCGCCTTCT
Ac33	GCCATGGCGAGAACAATTTGCAGCTGGGTTTCCCTACTCCCATTTGCCC[A/G]ACAATAATCACCATCCTTCAAAGCCCGCCTTCTGCAGCTCAGGGCAGTTCT
Ac34	CTGCAGCTCAGGGCAGTTCTTCAGAGGTCCATCTTTTCTATAGAGTGAC[T/C]GGCTTTCGAAATCTTGAACTTTATGAGAGGGCCTCTCTGTCTTACATCAAA
Ac36	TCATTTCTAGCACCCCATACTTTCGCGGTAGAATGCGTTTCTGGTTCAT[T/C]CCAACTTATATTGGGGATGAGTATCGTACTCTGGATGTTGCCTATCTGAAC
Ac37	CATTTGGTCTTACCAAAATGGACAGCAGCTTCCAGAGAACTATGTACGG[G/A]GGTCTGGTCGTCGGACATAGCACACGCGGAGAACCAATTGCTCTGGACGT
Ac38	CAGCAGCTTCCAGAGAACTATGTACGGAGGTCTGGTCGTCGGACATAG[T/C]ACACGCGGAGAACCAATTGCTCTGGACGTCCCTGCTAGACACATCTTTTC
Ac40	CGTGGGCTCCCCACAGTCATGTGACGCATAAGATGATGCAACAAACCAC[T/C]GGAGAGCAGTACTAAGGGTCACAGTAATCCTATTTAATGAGCTAGCGATGT
Ac41	TGTGACGCATAAGATGATGCAACAAACCACCGGAGAGCAGTACTAAGGG[C/T]CACAGTAATCCTATTTAATGAGCTAGCGATGTTATCCTTACATCTTTTTAT
Ac42	ATCAAGCATACCAAGGCGATTGCGCCGCCAACAGCTGGCCCAATTCAGC[A/T]TACATTACTGGTTATTCATATGTGCGAAGCAACGACGAAAGCAGCATGAAG
Ac43	CCAGTGGAGACAACTTTCAATATTACAATGGCGGTGTGTTTAGTGGACC[T/C]TGTGGAACTAGTCTCAATCATGCCATCACCATTATAGGTTACGGGCAGGAT
Ac44	TTGGATTGTAAAGAACTCATGGGGTAGCTCATGGGGTGAACGTGGATAC[G/A]TCCGTATGGCGAGAGGTGTGTCTTCGTCTGGATTATGTGGAATCGCCATGG
Ac45	TGGCCTTTGTTCTGTGAAAAATCTCTCCTTTTCTTTGATCTGTTTTTCG[A/G]CCGTGTTAGGAAGGGTTAGATAAGATGATGTCTCTCGTATTGTTGGCCTGT
Ac46	GGTTAGATAAGATCATGTCTCCCGTATTGTTGACTTGTAATCTTATTGT[A/G]TTTTCAACACAATTTTATGTGTCCTTAGTGGTGTAAAGCGCAAATAAATAA
Ac47	ACCCTAAGATACATTATGAGACAACTGGACCTGAAATTTGGGAAGGCAC[A/G]GGGCACAAAATTGACGGCCTTGTTTCTGGTATTGGAACTGGCGGCACGATC
Ac48	GCCGAGGGTACGTGGAGGACGTGAGGCTGAGCAACGTGAGGCTGCTGAT[C/T]GGATCCATCATCATCGCCATCGCGCTTCTCGCCCAATTCTACCCCAAGAAG
Ac50	GCAGCACTGGTGCTGCAAAGGCTGTTGGCAAGGTGCTTCCTGCTTTGAA[T/C]GGCAAGTTGACTGGTATGGCTTTCCGTATTCCTACTGTTGATGTCTCCGTC
Ac51	TGAGGGAAAACTTAAAGGAATTCTAGGTTATGTAGACGAGGACTTGGT[T/C]TCCTCAGACTTTGTGGGTGACAGCAGGTCAAGCATCTTTGATGCCAGGGCT
Ac53	CACAGTGATAATTCCGCAGTGGTAAACACTATGGCAGAAAATGGACGGC[T/C]GGTGAATGATTTCCAATTTGGTCCAGAGTATAAAGATATGACGGCGTTGCT
Ac54	TAGTAGAGATGGGGAGAGGGAGAGTTGAGCTGAAGAGGATCGAGAACAA[A/G]ATCAACCGGCAAGTGACGTTCTCGAAGCGCCGCAACGGGCTCCTCAAGAAG
Ac55	GAAATCGAGGTACTGCCGTGGTGTTCCTGACCCAAAGATCCGTATCTA[C/T]GATGTTGGTATGAAGAAGAAGGGAGTCGATGAGTTCCCCTTCTGCGTCCAT
Ac56	GTATCTACGATGTTGGTATGAAGAAGAAGGGAGTCGATGAGTTCCCCTT[C/T]TGCGTCCATTTAGTAAGCTGGGAGAAAGAAAATGTTTCTAGTGAAGCTCTT
Ac58	ATGCCGGGAAGGATGCTTTCCATCTTAGGGTTAGGGTTCACCCTTTCCA[T/C]GTTCTTCGGATCAACAAGATGCTTTCCTGTGCTGGGGCTGATCGGCTCCAA
Ac59	ATAGTAACAGCAATCACGCCCAAGAAGCGCTTCGCCGTGCCAAGTTCAA[G/A]TTCCCTGGTCGTCAAAAGATCATCATCAGCAGGAAATGGGGATTCACTAAA
Ac60	GTGCCAAGTTCAAATTCCCTGGTCGTCAAAAGATCATCATCAGCAGGAA[G/A]TGGGGATTCACTAAATTTAGCCGCACTGACTATCTGAAGTGGAAAAGCGAG
Ac62	ACAAGCTTTCCTGCCAGAGACAATTGCAGATGCCTCTTAGATCATGTTC[C/T]GTGGAGAAGCACTATCAATAGCTTTTGGTTCTTTAAAGTCTTTAGTACTTG
Ac63	TTTTCCTAACAGACAAAATCTCCTTCTGTGTTCCTCGTTTTTGTTTGTC[G/A]AACTGCGCCGCGTGATCTTGTATGCTTGAACTGCTATGTTGGTCGGGGTA
Ac70	AAAACCCCTGTGGTTCTTGCTGGGCATTCGCTGCAATTGCGACGGTGGA[A/G]GGAATCTACAAGATCAAAACAGGGTACTTAGTATCTTTATCGGAGCAAGAA
Ac73	GGATAGTAAGGAACTCGTGGGGCAGCTCATGGGGTGAGGGTGGATACGT[T/C]CGTATGGCAAGAGGTGTGTCATCGTCATCTGGAGTATGTGGAATCGCCAT
Ac75	ATGGCAAGAGGTGTGTCATCGTCATCTGGAGCATGTGGAATCGCCATG[T/G]CTCCTCTCTTTCCCACTCTACAATCAGGGGCTAATGTCGAAGTTATTAAGA
Ac76	GGAATCGCCATGGCTCCTCTCTTTCCCACTCTACAATCAGGGGCTAATG[T/C]CGAAGTTATTAAGATGGTTTCTGAAACTTGAAGCTACGTAATAAGTCGGAA
Ac77	TTTGCGCACTTATGTCATAAAAGTATGTGTCTGTGATGGTGTGTTTGTG[A/G]TAGTAAGCCAAAGATCGGGCTTGGCTTCTATTCTATGGGTTTGATAGCATA
Ac81	AGGAGAATTCTATTGACATGGTTACAATCAACCCTGCTATGGTCATAGG[T/C]CCTCTTTTGCAGCCTACTCTCAACACGAGCTGTGAGGCAATCCTCAAATTA
Ac82	AGATGCGCCGGTTTCAGATATTCAAGAACAACGTGAACCATATCGAAAC[C/T]TTTAACAGTCGCAATAAAAATTCGTACACTCTCGGCATCAATCAGTTTACC
Ac84	CTAAAGGCTACGGGTGCAAAGGCGGCTGGGAGTTCAGGGCCTTCGAATT[C/T]ATCATATCTAACAAGGGCGTGGCATCGGGAGCTATCTACCCTTACAAAGCA
Ac85	GAGTTCAGGGCCTTCGAATTCATCATATCTAACAAGGGCGTGGCATCGG[G/C]AGCTATCTACCCTTACAAAGCAGCCAAAGGCACCTGCAAGACCAATGGCGT
Ac94	AGGAGCCACATGCCCAGGGGTCTGCGTGTGCCACAGCAGGTTTTTCTAA[T/C]GGCAGTAGGATTTTAACAGTGGTGCCACATGATGGGGAGTTGGACAATATT
Ac95	CCACAGCAGGTTTTTCTAACGGCAGTAGGATTTTAACAGTGGTGCCACA[C/T]GATGGGGAGTTGGACAATATTCTGGTTATGCCAGCCTCCCCAGCAATTCT
Ac96	CGGAATCGCGCAATATGGTGCAAATGAAGCTAACACCAGGCTCAATTTT[T/C]CGAGATCAAGCTTCCTTGCTAGCAGTACTCATTTCTAGCACCCCATACTTT
Ac97	GGCACAGAGTGCAATTTGAATACTTGAGGACTCGATACTTGATCTCCCA[T/C]ATCACGGTGAACAGCAAGACACAGCCTGGGACAACTTACAAAACTTTGCTG
Ac100	TGAACAGCAAGACACAGCCTGGGACAACTTACAAAACTTTGCTGCGTCC[C/T]GCCATTGGTTATGTTTTTGATAAAGGGAGAAAGTCTTCAAAGAACACCAGC
Ac101	CAAAGAATACCAGCGCAGCTCTGTTTTCCCACTATAACTTCTGGAACGC[T/C]ACTGTGGAGATGGAAATACTACTCTCGAGTAGGCGGGGAGTTGCAGGAACA
Ac102	AGGTCTGGTCGTCGGACATAGCACACGCGGAGAACCAATTGCTCTGGA[T/C]GTCCCTGCTAGACACATCTTTTCACGATCCAGGATTCGCCACAAGGACAA
Ac104	TCAGCAGTTCAGGATGCCAGACCTTAGCACTGTGATGGCCAAGCCCGAT[G/A]CGTCTGCTACAGCCGCAGCGGATGAAGAAGAGGAGGATATCGATGAAACTG
Ac105	ATCAAACATTTACATTCTCTGGGCCAACCAAACTGAATATACACTTTGA[C/T]AATTACGCCTGGTTGGGAGGGAGTCTGTGCCATGGCGAGAACAATTTGCAG

**Table 3:** Examples of DNA fingerprints based on the array of 57 SNPs for pineapple genotype identification (showing truncated profiles).

		Ac28	Ac30	Ac41	Ac59	Ac84	Ac89	Ac15	Ac48	Ac50	Ac53	Ac56	Ac64	Ac65	Ac78	Ac92	Ac20	Ac5	Ac6	Ac91	Ac13	Ac2	Ac22	Ac54	Ac66	Ac68	Ac44	Ac85	Ac58	Ac63	Ac76	Ac16	Ac46	Ac55	Ac23
PERNAMBUCO	Singapore	A G	C C	C T	A G	C C	A A	A A	C C	C C	C T	C T	C C	A A	G G	A G	G G	A T	G T	T T	A G	T T	A T	G G	C C	G G	A A	G G	C T	A G	C T	C T	G G	C T	C T
ABACAXI	Brazil	A G	C C	C T	A G	C C	A A	A A	C C	C C	C T	C T	C C	A A	G G	A G	0 0	A T	G T	T T	A G	T T	A T	G G	C C	G G	A A	G G	C T	A G	C T	C T	G G	C T	C T
SUGAR LOAF	Zaire	A G	C C	C T	A G	C C	A A	A A	C C	C C	C T	C T	C C	A A	G G	A G	G G	A T	G T	T T	A G	T T	A T	G G	C C	G G	A A	G G	C T	A G	C T	C T	G G	C T	C T
BERMUDA	Barbados	A A	A C	C T	G G	C C	A A	A A	C T	C C	C T	C C	A C	G G	G G	A A	A G	A T	G T	T T	A A	A T	A T	A G	C G	C C	A A	G G	T T	A G	C T	C T	G G	C T	C T
REDONDA	Venezuela	A A	A C	C T	G G	C C	A A	A A	C T	C C	C T	C C	A C	G G	G G	A A	A G	A T	G T	T T	A A	A T	A T	A G	C G	C C	A A	G G	T T	A G	C T	C T	G G	C T	C T
SARAWAK	Malaysia	A A	A C	C T	G G	C C	A A	A A	C T	C C	C T	C C	A C	G G	G G	A A	G G	A T	G T	T T	A A	A T	A T	A G	C G	C C	A A	G G	T T	A G	C T	C T	G G	C T	C T
MONTE LIRIO	Guatemala	A A	C C	T T	A A	C C	A G	A A	C T	C C	C C	T T	C C	A A	G G	A G	G G	A A	T T	T T	A A	T T	A A	G G	C C	G G	A A	G G	C C	A G	C T	C T	G G	C T	T T
CAMBRAY	Philippines	A A	C C	T T	A A	C C	A G	A A	C T	C C	C C	T T	C C	A A	G G	A G	G G	A A	T T	T T	A A	T T	A A	G G	C C	G G	A A	G G	C C	A G	C T	C T	G G	C T	T T
JANDAIRA	Brazil	A A	A C	C T	A G	C T	A G	C C	C C	C C	C C	C T	C C	A A	G G	A G	G G	A T	G T	T T	A G	A T	A A	G G	C C	G G	A G	C G	C C	A G	C T	C T	G G	C T	C T
REZENDE	Brazil	A A	A C	C T	A G	C T	A G	C C	C C	C C	C C	C T	C C	A A	G G	A G	G G	A T	G T	T T	A G	A T	A A	G G	C C	G G	A G	C G	C C	A G	C T	C T	G G	C T	C T

**Table 4:** List of 24 synonymous groups, including 130 accessions, identified by SNP markers in USDA pineapple collection, Hilo, Hawaii.

Grp	Code	Name	
1	HANA 10	Cayenne Hilo	Hawaii, USA
1	HANA 100	Cayenne #59 4N	Hawaii, USA
1	HANA 111	Cayenne M111 Seedy Fruit	Hawaii, USA
1	HANA 112	Cayenne Paper Leaf	Hawaii, USA
1	HANA 114	Cayenne Bottleneck	Hawaii, USA
1	HANA 115	Cayenne M226 Nubby	Hawaii, USA
1	HANA 119	Cayenne M267 Dry Sweet	Hawaii, USA
1	HANA 133	Kew	Philippines
1	HANA 92	Cayenne 573	Hawaii, USA
1	HANA 94	Cayenne Clone 9	Hawaii, USA
1	HANA 95	Cayenne 1069	Hawaii, USA
1	HANA 99	Cayenne #31 4N	Hawaii, USA
1	N90-92	N90-92	Hawaii, USA
1	HANA 179	3621 N03-23	Hawaii, USA
1	HANA 162	Cayenne John Teves	Hawaii, USA
1	HANA 101	Cayenne M 4W	Hawaii, USA
1	HANA 167	32419 N91-15	Florida, USA
1	HANA 103	Cayenne M 61 Low Bloom	Hawaii, USA
1	HANA 104	Cayenne M 63 Plus Bloom	Hawaii, USA
1	HANA 110	Cayenne M109-5	Hawaii, USA
1	HANA 117	Cayenne M 35	Hawaii, USA
1	HANA 123	Red Spanish	Balboa, Panama
1	HANA 170	N91-34	Hawaii, USA
1	HANA 178	Chimpaka N03-22	Hawaii, USA
1	HANA 93	Cayenne 666	Hawaii, USA
1	HANA 102	Cayenne M 24	Hawaii, USA
1	HANA 105	Cayenne M 91 Big Eye Type Big Eye #3	Hawaii, USA
1	HANA 106	Cayenne M92 Big Eye John Johnson 4395	Hawaii, USA
1	HANA 107	Cayenne M105 Big Eye Excess	Hawaii, USA
1	HANA 108	Cayenne Seedy #24	Hawaii, USA
1	HANA 116	Cayenne CPC Big Eye	Hawaii, USA
1	HANA 139	Cayenne Azores	Azores, Portugal
1	HANA 163	N91-05	Chiang Rai, Thailand
1	HANA 180	153 N03-24	Hawaii, USA
1	HANA 98	Cayenne 45 #5 4N	Hawaii, USA
1	HANA 176	N00-10 Dole Cayenne	Hawaii, USA
			
2	HANA 53	British Samoa P1	British Samoa
2	HANA 11	Columbia Variety No. 1	Colombia
2	HANA 54	British Samoa P5	British Samoa
2	HANA 55	Apaporis	Apaporis, Colombia
2	HANA 56	Apaporis P1	Apaporis, Colombia
2	HANA 62	Rio Kananari	Rio Kananari, East Colombia
3	HANA 13	Spanish Samoa	American Samoa
3	HANA 17	Natal	Natal, South Africa
3	HANA 24	Montserrat	Philippines
3	HANA 25	Macgregor	Philippines
3	HANA 28	Dacca	Philippines
3	HANA 164	N91-06	Chumporn, Thailand
4	HANA 14	Pernambuco	Singapore
4	HANA 46	Sugar Loaf	Zaire
4	HANA 21	Abacaxi	Brazil
5	HANA 16	Bermuda	Barbados, BWI
5	HANA 137	Redonda	Pantanillo, Venezuela
5	HANA 19	Sarawak	Malaysia
			
6	HANA 18	Mauritius	Taiwan
6	HANA 29	Sugar Loaf	Philippines
6	HANA 125	Jamaica Sugar	Jamaica, WI
6	HANA 23	Sam Clarke Plot	West Indies, Jamaica
6	HANA 43	Mo	Vietnam
6	HANA 26	Philippine Red	Philippines
6	HANA 30	Sylhet Jaldubi	Philippines
6	HANA 33	Ananas Kendal	Java, Indonesia
6	HANA 41	Pho Lang Tuang	Vietnam
6	HANA 42	Saigon Red	Vietnam
6	HANA 44	Moe	Vietnam
6	HANA 122	Uhi	Taiwan
6	HANA 127	Kohi	Taiwan
6	HANA 134	Kumta	Karnataka, India
6	HANA 154	CB 67	Brazil
6	HANA 128	Spiny Anpi	Taiwan
6	HANA 129	Philippine Green	Philippines
7	HANA 37	Criolla	Guadalajara Jalisco, Mexico
7	HANA 60	Spanish Guatemala	Guatemala
7	HANA 48	Mexican Criolla	Tamazunchale, Mexico
8	HANA 64	CB 5	Tatuhy, Brazil
8	HANA 68	CB 11	Mogy-Mirim, Brazil
9	HANA 12	Congo	Africa
9	HANA 15	Ruby	Singapore
9	HANA 27	Wild Kailua	Manoa, Hawaii, USA
9	HANA 31	Black Antigua	Philippines
9	HANA 35	Amalsad	Gujarat, India
9	HANA 45	Nep	Vietnam
9	HANA 71	CB 18	Puerto Bertoni, Paraguay
9	HANA 78	CB 36	Dourados, Brazil
9	HANA 85	Fazenda Moura	Rio de Janeiro, Brazil
9	HANA 90	Prazeres	Prazeres, Brazil
9	HANA 118	ACC. 253	Hawaii, USA
9	HANA 120	Los Banos	Hawaii, USA
9	HANA 130	Klajatan	Buitenzorg, Java, Indonesia
9	HANA 131	Ananas Merah	Java, Indonesia
9	HANA 140	Pakse	Vietnam
9	HANA 142	Den	Vietnam
9	HANA 148	CB 24	San Lorenzo, Paraguay
9	HANA 149	CB 33	Bella Vista, Brazil
9	HANA 153	CB 65	Rio de Janeiro, Brazil
10	HANA 145	Cabezona	Puerto Rico
10	HANA 165	N91-13 31358	Florida, USA
11	HANA 146	Antigua	Antigua, Guatemala
11	HANA 121	Amarillo	Brazil
11	HANA 150	CB 38	Ponta Pora, Brazil
12	HANA 20	Plot 347 (PI 56907)	Brazil
12	HANA 73	CB 20	Puerto Bertoni, Paraguay
12	HANA 74	CB 21	Puerto Bertoni, Paraguay
12	HANA 185	N04-8	Brazil
12	HANA 75	CB 23	San Ignacio, Argentina
13	HANA 3	61-2223 (PI 536881)	Hawaii, USA
13	HANA 61	Unknown Foreign Variety	N/A
14	HANA 86	Jandaira	Dodora, Brazil
14	HANA 87	Rezende	Rezende, Brazil
15	HANA 96	Cayenne 7898 Qc	Hawaii, USA
15	HANA 97	Cayenne 7898 4n	Hawaii, USA
16	HANA 172	N94-92 Short Fruit #1 NGRL 33	Santa Cruz, Bolivia
16	HANA 174	N94-92 Long Fruit #1 NGRL 33	Santa Cruz, Bolivia
16	HANA 173	N94-92 Short Fruit #2 NGRL 33	Santa Cruz, Bolivia
17	HANA 136	Spanish Criolla Red	El Hatillo, Venezuela
17	HANA 138	Red Spanish Pina Lisa	Pantanillo, Venezuela
18	HANA 161	58-474 (PI 536979)	Hawaii, USA
18	HANA 158	57-503 (PI 536977)	Hawaii, USA
19	HANA 51	Ananas De Vaupes	Bogota, Colombia
19	HANA 169	32424 N91-17	Florida, USA
20	HANA 63	CB 2	Rio de Janeiro, Brazil
20	HANA 66	CB 9	Amazon, Brazil
21	HANA 81	Rondon	Campinas, Brazil
21	HANA 39	Philippine Hybrid	Philippines
22	HANA 34	Monte Lirio	Guatemala
22	HANA 32	Cambray	Philippines
23	HANA 50	Bogota	Bogota, Colombia
23	HANA 67	CB 10	Mogy-Mirim, Brazil
24	HANA79	F1 Hybrid Campinas	Campinas, Brazil
24	HANA80	F1 Hybrid var. *ananassoides* X Rondon	Hawaii, USA

**Table 5:** Minor allele frequency, information index, heterozygosity, and inbreeding coefficient of the 57 SNP loci scored on 64 pineapple accessions.

SNP ID	Minor allele frequency	Information index	Observed heterozygosity	Expected heterozygosity	Inbreeding coefficient
Ac4	0.440	0.686	0.433	0.493	0.122
Ac5	0.313	0.622	0.478	0.430	−0.110
Ac6	0.313	0.622	0.388	0.430	0.098
Ac9	0.159	0.438	0.288	0.268	−0.076
Ac10	0.239	0.550	0.478	0.364	−0.314
Ac11	0.246	0.558	0.284	0.371	0.236
Ac13	0.440	0.686	0.433	0.493	0.122
Ac15	0.379	0.663	0.485	0.471	−0.030
Ac17	0.366	0.657	0.433	0.464	0.067
Ac19	0.119	0.366	0.239	0.210	−0.136
Ac20	0.431	0.684	0.800	0.490	−0.631
Ac21	0.254	0.566	0.507	0.379	−0.340
Ac31	0.067	0.246	0.134	0.125	−0.072
Ac32	0.396	0.671	0.791	0.478	−0.654
Ac33	0.418	0.680	0.836	0.487	−0.718
Ac34	0.090	0.302	0.060	0.163	0.634
Ac36	0.336	0.638	0.433	0.446	0.030
Ac37	0.357	0.652	0.429	0.459	0.067
Ac38	0.100	0.325	0.015	0.180	0.915
Ac40	0.157	0.434	0.254	0.264	0.040
Ac41	0.119	0.366	0.239	0.210	−0.136
Ac42	0.269	0.582	0.418	0.393	−0.063
Ac43	0.362	0.654	0.723	0.462	−0.566
Ac44	0.453	0.689	0.438	0.496	0.117
Ac45	0.112	0.351	0.194	0.199	0.024
Ac46	0.265	0.578	0.470	0.390	−0.205
Ac47	0.300	0.611	0.600	0.420	−0.429
Ac48	0.396	0.671	0.433	0.478	0.095
Ac50	0.396	0.671	0.433	0.478	0.095
Ac51	0.119	0.366	0.239	0.210	−0.136
Ac53	0.400	0.673	0.800	0.480	−0.667
Ac54	0.077	0.271	0.154	0.142	−0.083
Ac55	0.321	0.628	0.463	0.436	−0.062
Ac56	0.410	0.677	0.403	0.484	0.167
Ac58	0.227	0.536	0.303	0.351	0.137
Ac59	0.302	0.612	0.349	0.421	0.171
Ac60	0.439	0.686	0.879	0.493	−0.784
Ac62	0.261	0.574	0.254	0.386	0.343
Ac63	0.127	0.380	0.254	0.222	−0.145
Ac70	0.276	0.589	0.493	0.400	−0.232
Ac73	0.336	0.638	0.672	0.446	−0.506
Ac75	0.402	0.674	0.803	0.481	−0.671
Ac76	0.216	0.522	0.433	0.339	−0.276
Ac77	0.470	0.691	0.463	0.498	0.071
Ac81	0.313	0.622	0.388	0.430	0.098
Ac82	0.455	0.689	0.879	0.496	−0.772
Ac84	0.493	0.693	0.448	0.500	0.104
Ac85	0.468	0.691	0.841	0.498	−0.689
Ac94	0.119	0.366	0.179	0.210	0.148
Ac95	0.333	0.637	0.667	0.444	−0.500
Ac96	0.052	0.205	0.104	0.099	−0.055
Ac97	0.354	0.650	0.708	0.457	−0.548
Ac100	0.452	0.689	0.841	0.495	−0.698
Ac101	0.306	0.616	0.484	0.425	−0.138
Ac102	0.423	0.681	0.846	0.488	−0.733
Ac104	0.067	0.246	0.104	0.125	0.166
Ac105	0.462	0.690	0.924	0.497	−0.859
Mean	0.298	0.565	0.465	0.385	−0.157

**Table 6:** Likelihood assignment of parentage of pineapple (*A. comosus* var. *comosus*) accessions based on 57 SNP markers with LOD scores above 80% probability.

Candidate parent^a^	Botanical variety	Offspring	LOD score^b^
CB 15	var. *ananassoides*	Wild Brazil x Lot 520	2.88
CB 15	var. *ananassoides*	Pina Lisa	1.25
CB 17	var. *bracteatus*	Canterra	2.84
CB 17	var. *bracteatus*	Papuri Vaupes Colombia	2.90
CB 17	var. *bracteatus*	CB 30	3.89
CB 17	var. *bracteatus*	Pina de Castilla	2.62
CB 19	var. *ananassoides*	Cayenne Lot 520	3.05
CB 5	var. *bracteatus*	Rondon	1.43
CB 6	var. *ananassoides*	Bogota	18.66
CB 61	var. *ananassoides*	CB 32	2.11
Plot 347 (PI 56907)	var. *bracteatus*	Congo	2.65
Plot 347 (PI 56907)	var. *bracteatus*	Phu Qui	2.84
Plot 347 (PI 56907)	var. *bracteatus*	Mauritius	2.31
Plot 347 (PI 56907)	var. *bracteatus*	Cheese Pine	0.61

a Putative parental accessions used in the present analysis. Only those with significant LOD score were listed.

b Critical LOD (the natural logarithm of the likelihood) ratio for assignment of parentage is 2.60 at 95% confidence and 0.24 at 80% confidence.

## References

[bib1] Food and Agriculture Organization of the United Nations. Statistical databases. United Nations: FAO, 2014. Available at http://faostat.fao.org/site/567/DesktopDefault.aspx?PageID=567#ancor (accessed December 31, 2014).

[bib2] Baker KF, Collins JL. Notes on the distribution and ecology of *Ananas* and *Pseudananas* in South America. Am J Bot 1939; 26: 697–702.

[bib3] Pearsall DM. The origins of plant cultivation in South America. In: Cowan CW, Watson PJ, editors. The Origins of Agriculture: An International Perspective. Washington, DC: Smithsonian Institution Press; 1992, pp 173–205.

[bib4] Bartholomew DP, Paull RE, Rohrbach KG. The Pineapple: Botany, Production and Uses. Wallingford: CABI Publishing; 2003.

[bib5] Purseglove JW. Tropical Crops. Monocotyledons. London: Longman; 1972. pp 75–91.

[bib6] Loison-Cabot C. Origin, phylogeny and evolution of pineapple species. Fruits 1992; 47: 25–32.

[bib7] Coppens d'Eeckenbrugge G, Leal F. “Chapter 2: Morphology, Anatomy, and Taxonomy”. In: Bartholomew DP, Paull RE, Rohrbach KG, editors. The Pineapple: Botany, Production, and Uses. Wallingford: CABI Publishing; 2003. pp 13–32.

[bib8] Coppens d’Eeckenbrugge G, Sanewski GM, Smith MK et al. *Ananas*. In: Kole C editor. Wild Crop Relatives: Genomic and Breeding Resources, Tropical and Subtropical Fruits. Berlin and Heidelberg: Springer-Verlag; 2011, pp 21–41.

[bib9] Morrison SE. Journals and Other Documents of the Life and Voyages of Christopher Columbus. New York: Heritage Press; 1973.

[bib10] Collins JL. The Pineapple, Botany, Utilisation, Cultivation. London: Leonard Hill Ltd; 1960. p294.

[bib11] Bartholomew DP, Hawkins RA, Lopez JA. Hawaii pineapple: the rise and fall of an industry. HortScience 2012; 47: 1390–1398.

[bib12] Hidayat T, Abdullah FI, Kuppusamy C, Samad AA, Wagiran A. Molecular identification of Malaysian pineapple cultivar based on internal transcribed spacer region. APCBEE Procedia 2012; 4: 146–151.

[bib13] Sripaoraya S, Marchant R, Power JB, Davey MR. Herbicide-tolerant transgenic pineapple (*Ananas comosus*) produced by microprojectile bombardment. Ann Bot 2001; 88: 597–603.

[bib14] Zhang J, Liu J, Ming R. Genomic analyses of the CAM plant pineapple. J Exp Bot 2014; 65: 3395–3404. 24692645 10.1093/jxb/eru101

[bib15] Aradhya MK, Zee F, Manshart RM. Isozyme variation in cultivated and wild pineapple. Euphytica 1994; 79: 87–99.

[bib16] Noyer JL, Lanaud C, Duval MF, Coppens G. RFLP study on rDNA variability in *Ananas* genus. Acta Hort 1997; 425: 153–160.

[bib17] Duval MF, Noyer JL, Perrier X, Coppens d’Eeckenbrugge G, Hamon P. Molecular diversity in pineapple assessed by RFLP markers. Theor Appl Genet 2001; 1: 83–90.

[bib18] Duval MF, Buso GSC, Ferreira FR et al. Relationships in *Ananas* and other related genera using chloroplast DNA restriction site variation. Genome 2003; 46: 990–1004. 14663518 10.1139/g03-074

[bib19] Popluechai S, Onto S, Eungwanichayapant PD. Relationships between some Thai cultivars of pineapple (*Ananas comosus*) revealed by RAPD analysis. Songklanakarin J Sci Technol 2007; 29: 1491–1497.

[bib20] Kato CY, Nagai C, Moore PH et al. Intra-specific DNA polymorphism in pineapple (*Ananas comosus* (L.) Merr.) assessed by AFLP markers. Genet Resour Crop Evol 2005; 51: 815–825.

[bib21] Wöhrmann T, Weising K. In silico mining for simple sequence repeat loci in a pineapple expressed sequence tag database and cross-species amplification of EST-SSR markers across Bromeliaceae. Theor Appl Genet 2011; 123: 635–647. 21625993 10.1007/s00122-011-1613-9

[bib22] Shoda M, Urasaki N, Sakiyama S et al. DNA profiling of pineapple cultivars in Japan discriminated by SSR markers. Breeding Sci 2012; 62: 352–359. 10.1270/jsbbs.62.352 PMC3528333 23341750

[bib23] Feng S, Tong H, Chen Y et al. Development of pineapple microsatellite markers and germplasm genetic diversity analysis. BioMed Res Int 2013; 2013: 11. 10.1155/2013/317912 PMC3760190 24024187

[bib24] Ji K, Zhang D, Motilal LA, Boccara M, Lachenaud P, Meinhardt LW. Genetic diversity and parentage in farmer varieties of cacao (*Theobroma cacao* L.) from Honduras and Nicaragua as revealed by single nucleotide polymorphism (SNP) markers. Genet Resour Crop Evol 2013; 60: 441–453.

[bib25] Cabezas JA, Ibáñez J, Lijavetzky D et al. A 48 SNP set for grapevine cultivar identification. BMC Plant Biol 2011; 11: 153. 22060012 10.1186/1471-2229-11-153 PMC3221639

[bib26] Wu GA, Prochnik S, Jenkins J et al. Sequencing of diverse mandarin, pummelo and orange genomes reveals complex history of admixture during citrus domestication. Nature Biotechnol 2014; 32: 656–662. 24908277 10.1038/nbt.2906 PMC4113729

[bib27] Ge AJ, Han J, Li XD et al. Characterization of SNPs in strawberry cultivars in China. Genet Mol Res 2013; 12: 639–645. 23546945 10.4238/2013.March.7.2

[bib28] Koia JH, Moyle R, Botella JR. Microarray analysis of gene expression profiles in ripening pineapple fruits. BMC Plant Biol 2012; 12: 240. 23245313 10.1186/1471-2229-12-240 PMC3568034

[bib29] Moyle R, Fairbairn DJ, Ripi J et al. Developing pineapple fruit has a small transcriptome dominated by metallothionein. J Exp Bot 2005; 56: 101–112. 15520025 10.1093/jxb/eri015

[bib30] Neuteboom LW, Kunimitsua WY, Webb D, Christopher DA. Characterization and tissue-regulated expression of genes involved in pineapple (*Ananas comosus* L.) root development. Plant Sci 2002; 5: 1021–1035.

[bib31] Ong WD, Voo L-YC, Kumar VS. *De Novo* assembly, characterization and functional annotation of pineapple fruit transcriptome through massively parallel sequencing. PLoS One 2012; 7: e46937. 23091603 10.1371/journal.pone.0046937 PMC3473051

[bib32] Tang J, Vosman B, Voorrips RE, van der Linden CG, Leunissen JA. QualitySNP: a pipeline for detecting single nucleotide polymorphisms and insertions/deletions in EST data from diploid and polyploid species. BMC Bioinformatics 2006; 7: 438. 17029635 10.1186/1471-2105-7-438 PMC1618865

[bib33] Zhang HN, Wei YZ, Shen JY et al. Transcriptomic analysis of floral initiation in litchi (*Litchi chinensis* Sonn.) based on de novo RNA sequencing. Plant Cell Rep 2014; 33: 1723–1735. 25023873 10.1007/s00299-014-1650-3

[bib34] Platel RK, Jain M. NGS QC Toolkit: a toolkit for quality control of next generation sequencing data. PLoS One 2012; 7: e30619. 22312429 10.1371/journal.pone.0030619 PMC3270013

[bib35] Wang J, Lin M, Crenshaw A et al. High-throughput single nucleotide polymorphism genotyping using nanofluidic dynamic arrays. BMC Genomics 2009; 10: 561. 19943955 10.1186/1471-2164-10-561 PMC2789104

[bib36] Fluidigm. Fluidigm SNP Genotyping User Guide Rev H1, PN 68000098. South San Francisco, CA: Fluidigm Corporation; 2011.

[bib37] Peakall R, Smouse PE. Genalex 6: Genetic analysis in excel. Population genetic software for teaching and research. Mol Ecol Notes 2006; 6: 288–295. 10.1093/bioinformatics/bts460 PMC3463245 22820204

[bib38] Peakall R. Smouse PE. GenAlEx 6.5: Genetic analysis in excel. Population genetic software for teaching and research-an update. Bioinformatics 2012; 8: 2537–2539. 10.1093/bioinformatics/bts460 PMC3463245 22820204

[bib39] Dieringer D, Schlötterer C. Microsatellite analyser (MSA): Aplatform independent analysis tool for large microsatellite datasets. Mol Ecol Notes 2003; 3: 167–169.

[bib40] Saitou N, Nei M. The neighbor-joining method: a new method for reconstructing phylogenetic trees. Mol Biol Evol 1987; 4: 406–425. 3447015 10.1093/oxfordjournals.molbev.a040454

[bib41] Felsenstein J. PHYLIP-phylogeny inference package (version 3.2). Cladistics 1989; 5: 164–166.

[bib42] Letunic I, Bork P. Interactive tree of life v2: Online annotation and display of phylogenetic trees made easy. Nucleic Acids Res 2011; 39: W478–W478. 10.1093/nar/gkr201 PMC3125724 21470960

[bib43] Pritchard JK, Stephens M, Donnelly P. Inference of population structure using multilocus genotype data. Genetics 2000; 155: 945–959. 10835412 10.1093/genetics/155.2.945 PMC1461096

[bib44] Earl DA, vonHoldt BM. Structure Harvester: website and program for visualizing STRUCTURE output and implementing the Evanno method. Conserv Genet Resour 2012; 4: 359–361.

[bib45] Marshall TC, Slate J, Kruuk LEB, Pemberton JM. Statistical confidence for likelihood-based paternity inference in natural populations. Mol Ecol 1998; 7: 639–655. 9633105 10.1046/j.1365-294x.1998.00374.x

[bib46] Kalinowski ST, Taper ML, Marshall TC. Revising how the computer program CERVUS accommodates genotyping error increases success in paternity assignment. Mol Ecol 2007; 16: 1099–1106. 17305863 10.1111/j.1365-294X.2007.03089.x

[bib47] Huang X, Madan A. CAP3: A DNA sequence assembly program. Genome Res 1999; 9: 868–877. 10508846 10.1101/gr.9.9.868 PMC310812

[bib48] NoyerJL. Preliminary study of genetic diversity of the genus *Ananas* by RFLP. Fruits (France) 1991; 46: 372–375.

[bib49] Irish BM, Cuevas HE, Simpson SA et al. *Musa* spp. germplasm management: microsatellite fingerprinting of USDA-ARS national plant germplasm system collection. Crop Sci 2014; 54: 2140–2151.

[bib50] Zerega N, Wiesner-Hanks T, Ragone D et al. Diversity in the breadfruit complex (*Artocarpus*, Moraceae): Genetic characterization of critical germplasm. Tree Genet Genomes 2015; 11: 4.

[bib51] Gross BL, Volk GM, Richards C. Identification of “Duplicate” accessions within the USDA-ARS national plant germplasm system Malus collection. JASHS 2012; 5: 333–342.

[bib52] Marchant CJ. Chromosome evolution in the Bromeliaceae. Kew Bulletin 1967; 21: 161–168.

[bib53] Brown GK, Palací CA, Luther HE. Chromosome numbers in Bromeliaceae. Am J Bot 1997; 76: 85–88.

[bib54] Leal F, Soule J. Maipure, a new spineless group of pineapple cultivars. HortScience 1977; 12: 393–403.

[bib55] Wee YC, Thongtham MLC. *Ananas comosus* (L.) Merr. In: Verheij EWM, Coronel RE, editors. Plant Resources of South-East Asia No. 2 Edible Fruits and Nuts. Wageningen: The Netherlands, Pudoc; 1991. pp66–71.

[bib56] Coppens d'Eeckenbrugge G, Leal F, Duval MF. Germplasm resources of pineapple. Hort Rev 1997; 21: 133–175.

[bib57] Clement CR, Cristo-Araújo MD, Coppens D’Eeckenbrugge G, Pereira AA, Picanço-Rodrigues D. Origin and domestication of native amazonian crops. Diversity 2010; 2: 72–106.

