# Supplementary material for: Developing single nucleotide polymorphism markers for the identification of pineapple (Ananas comosus) germplasm
Source: Hortic Res. 2015 Nov 25;2:15056–. doi: 10.1038/hortres.2015.56 (PMC4660223; doi:10.1038/hortres.2015.56)
Supplement: Supplementary Table S1 [file hortres201556-s1.pdf]

Supplementary Table S1. List of 170 *Ananas comosus* accessions in USDA/ARS pineapple collection at Hilo, Hawaii.

Code	Species	Name of accessions
HANA1	*A. comosus* var. *comosus*	41-411
HANA2	*A. comosus* var. *comosus*	59-656
HANA3	*A. comosus* var. *comosus*	61-2223
HANA4	*A. comosus* var. *comosus*	58-1184 (PI 536882)
HANA6	*A. comosus* var. *comosus*	Tainung #9
HANA7	*A. comosus* var. *comosus*	Cayenne Lot 520 WBC
HANA8	*A. comosus* var. *comosus*	Cayenne Lanai
HANA10	*A. comosus* var. *comosus*	Cayenne Hilo
HANA11	*A. comosus* var. *comosus*	Columbia Variety No. 1
HANA12	*A. comosus* var. *comosus*	Congo
HANA13	*A. comosus* var. *comosus*	Spanish Samoa
HANA14	*A. comosus* var. *comosus*	Pernambuco
HANA15	*A. comosus* var. *comosus*	Ruby
HANA16	*A. comosus* var. *comosus*	Bermuda
HANA17	*A. comosus* var. *comosus*	Natal
HANA18	*A. comosus* var. *comosus*	Mauritius
HANA19	*A. comosus* var. *comosus*	Sarawak
HANA20	*A. comosus* var. *bracteatus*	Plot 347 (PI 56907)
HANA21	*A. comosus* var. *comosus*	Abacaxi
HANA22	*A. comosus* var. *comosus*	Santa Marta No. 1
HANA23	*A. comosus* var. *comosus*	Sam Clarke
HANA24	*A. comosus* var. *comosus*	Montserrat
HANA25	*A. comosus* var. *comosus*	Macgregor
HANA26	*A. comosus* var. *comosus*	Philippine Red
HANA27	*A. comosus* var. *comosus*	Wild Kailua
HANA28	*A. comosus* var. *comosus*	Dacca
HANA29	*A. comosus* var. *comosus*	Sugarloaf
HANA30	*A. comosus* var. *comosus*	Sylhet Jaldubi
HANA31	*A. comosus* var. *comosus*	Black Antigua
HANA32	*A. comosus* var. *comosus*	Cambray
HANA33	*A. comosus* var. *comosus*	Kendal
HANA34	*A. comosus* var. *comosus*	Monte Lirio
HANA35	*A. comosus* var. *comosus*	Amalsad
HANA36	*A. comosus* var. *comosus*	Cowboy
HANA37	*A. comosus* var. *comosus*	Criolla
HANA38	*A. comosus* var. *ananassoides*	Wild Brazil
HANA39	*A. comosus* hybrid	Philippine Hybrid
HANA40	*A. comosus* var. *comosus*	Phu Qui
HANA41	*A. comosus* var. *comosus*	Pho Lang Tuang
HANA42	*A. comosus* var. *comosus*	Saigon Red
HANA44	*A. comosus* var. *comosus*	Moe
HANA45	*A. comosus* var. *comosus*	Nep
HANA46	*A. comosus* var. *comosus*	Sugarloaf
HANA47	*A. comosus* var. *comosus*	Canterra
HANA48	*A. comosus* var. *comosus*	Mexican Criolla
HANA49	*A. comosus* var. *comosus*	Pina Criolla
HANA50	*A. comosus* var. *comosus*	Bogota
HANA51	*A. comosus* var. *comosus*	Ananas De Vaupes
HANA52	*A. comosus* var. *comosus*	Papuri Vaupes Colombia
HANA53	*A. comosus* var. *comosus*	British Samoa P1
HANA54	*A. comosus* var. *comosus*	British Samoa P5
HANA55	*A. comosus* var. *comosus*	Apaporis
HANA56	*A. comosus* var. *comosus*	Apaporis P1
HANA58	*A. comosus* hybrid	Wild Brazil X Lot 520
HANA59	*A. comosus* hybrid	F1 Cayenne X *P. sagenarius*
HANA60	*A. comosus* var. *comosus*	Spanish Guatemala
HANA61	*A. comosus* var. *comosus*	Unknown Foreign Variety
HANA62	*A. comosus* var. *comosus*	Rio Kanari
HANA63	*A. comosus* var. *erectifolius*	CB 2
HANA64	*A. comosus* var. *bracteatus*	CB 5
HANA65	*A. comosus* var. *comosus*	CB 6
HANA66	*A. comosus* var. *erectifolius*	CB 9
HANA67	*A. comosus* var. *ananassoides*	CB 10
HANA68	*A. comosus* var. *bracteatus*	CB 11
HANA69	*A. comosus* var. *ananassoides*	CB 15
HANA70	*A. comosus* var. *bracteatus*	CB 17
HANA71	*A. comosus* var. *comosus*	CB 18
HANA72	*A. comosus* var. *ananassoides*	CB 19
HANA73	*A. comosus* var. *bracteatus*	CB 20
HANA74	*A. comosus* var. *bracteatus*	CB 21
HANA75	*A. comosus* var. *bracteatus*	CB 23
HANA76	*A. comosus* var. *comosus*	CB 30
HANA77	*A. comosus* var. *comosus*	CB 32
HANA78	*A. comosus* var. *comosus*	CB 36
HANA79	*A. comosus* hybrid	F1 Hybrid Campinas
HANA80	*A. comosus* hybrid	F1 Hybrid var. *ananassoides* X Rondon
HANA81	*A. comosus* var. *comosus*	Rondon
HANA82	*A. comosus* var. *bracteatus*	Albus
HANA83	*A. comosus* var. *ananassoides*	CB 61
HANA85	*A. comosus* var. *comosus*	Fazenda Moura
HANA86	*A. comosus* var. *comosus*	Jandaira
HANA87	*A. comosus* var. *comosus*	Rezende
HANA88	*A. comosus* var. *ananassoides*	CB 71
HANA90	*A. comosus* var. *comosus*	Prazeres
HANA91	*A. comosus* var. *comosus*	Trinidad
HANA92	*A. comosus* var. *comosus*	Cayenne 573
HANA93	*A. comosus* var. *comosus*	Cayenne 666
HANA94	*A. comosus* var. *comosus*	Cayenne Clone 9
HANA95	*A. comosus* var. *comosus*	Cayenne 1069
HANA96	*A. comosus* var. *comosus*	Cayenne 7898 QC
HANA97	*A. comosus* var. *comosus*	Cayenne 7898 4N
HANA98	*A. comosus* var. *comosus*	Cayenne 45 No. 5 4N
HANA99	*A. comosus* var. *comosus*	Cayenne #31 4N
HANA100	*A. comosus* var. *comosus*	Cayenne #59 4N
HANA101	*A. comosus* var. *comosus*	Cayenne M 4W
HANA102	*A. comosus* var. *comosus*	Cayenne M 24
HANA103	*A. comosus* var. *comosus*	Cayenne M 61 Low Bloom
HANA104	*A. comosus* var. *comosus*	Cayenne M 63 Plus Bloom
HANA105	*A. comosus* var. *comosus*	Cayenne M 91 Big Eye
HANA106	*A. comosus* var. *comosus*	Cayenne M 92 Big Eye John
HANA107	*A. comosus* var. *comosus*	Cayenne M 105 Big Eye
HANA108	*A. comosus* var. *comosus*	Cayenne Seedy No. 24
HANA110	*A. comosus* var. *comosus*	Cayenne M 109-5
HANA111	*A. comosus* var. *comosus*	Cayenne M 111 Seedy Fruit
HANA112	*A. comosus* var. *comosus*	Cayenne Paper Leaf
HANA114	*A. comosus* var. *comosus*	Cayenne Bottleneck
HANA115	*A. comosus* var. *comosus*	Cayenne M 226 Nubby
HANA116	*A. comosus* var. *comosus*	Cayenne CPC Big Eye
HANA117	*A. comosus* var. *comosus*	Cayenne M 35
HANA118	*A. comosus* var. *comosus*	Acc. 253
HANA119	*A. comosus* var. *comosus*	Cayenne M 267 Dry Sweet
HANA120	*A. comosus* var. *comosus*	Los Banos
HANA121	*A. comosus* var. *comosus*	Amarillo
HANA122	*A. comosus* var. *comosus*	Uhi
HANA123	*A. comosus* var. *comosus*	Red Spanish
HANA124	*A. comosus* var. *comosus*	Taboga
HANA125	*A. comosus* var. *comosus*	Jamaica Sugar
HANA126	*A. comosus* var. *comosus*	Smooth Anpi
HANA127	*A. comosus* var. *comosus*	Kohi
HANA128	*A. comosus* var. *comosus*	Spiny Anpi
HANA129	*A. comosus* var. *comosus*	Philippine Green
HANA130	*A. comosus* var. *comosus*	Klajatan
HANA131	*A. comosus* var. *comosus*	Ananas Merah
HANA132	*A. comosus* var. *comosus*	Cheese Pine
HANA133	*A. comosus* var. *comosus*	Kew
HANA134	*A. comosus* var. *comosus*	Kumta
HANA136	*A. comosus* var. *comosus*	Spanish Criolla Red
HANA137	*A. comosus* var. *comosus*	Redonda Red Spanish

Code	Species	Name of accessions
HANA138	*A. comosus* var. *comosus*	Red Spanish Pina Lisa
HANA139	*A. comosus* var. *comosus*	Cayenne Azores
HANA140	*A. comosus* var. *comosus*	Pakse
HANA142	*A. comosus* var. *comosus*	Den
HANA143	*A. comosus* var. *comosus*	Pina De Castilla
HANA144	*A. comosus* var. *comosus*	Manzana
HANA145	*A. comosus* var. *comosus*	Cabezona
HANA146	*A. comosus* var. *comosus*	Antigua
HANA147	*A. comosus* var. *comosus*	Abacaxi Vermelho
HANA148	*A. comosus* var. *comosus*	CB 24
HANA149	*A. comosus* var. *comosus*	CB 33
HANA150	*A. comosus* var. *comosus*	CB 38
HANA151	*A. comosus* var. *comosus*	CB 46
HANA153	*A. comosus* var. *comosus*	CB 65
HANA154	*A. comosus* var. *comosus*	CB 67
HANA155	*A. comosus* var. *comosus*	White Jade
HANA156	*A. comosus* var. *comosus*	58-696 PI 536975
HANA157	*A. comosus* var. *comosus*	63-759 PI 536976
HANA158	*A. comosus* var. *comosus*	57-503 PI 536977
HANA160	*A. comosus* var. *comosus*	53-116 PI 536978
HANA161	*A. comosus* var. *comosus*	58-474 PI 536979
HANA162	*A. comosus* var. *comosus*	Cayenne John Teves
HANA163	*A. comosus* var. *comosus*	N91-05
HANA164	*A. comosus* var. *comosus*	N91-06
HANA165	*A. comosus* var. *comosus*	N91-13 31358
HANA166	*A. comosus* var. *comosus*	N91-14 27280
HANA167	*A. comosus* var. *comosus*	32419 N91-15
HANA168	*A. comosus* var. *comosus*	31722 N91-16
HANA169	*A. comosus* var. *comosus*	32424 N91-17
HANA170	*A. comosus* var. *comosus*	N91-34
HANA171	*A. comosus* var. *comosus*	75-50 N93-66
HANA172	*Ananas* species	N94-92 Short Fruit #1 NGRL 33
HANA173	*Ananas* species	N94-92 Short Fruit #2 NGRL 33
HANA174	*Ananas* species	N94-92 Long Fruit #1 NGRL 33
HANA175	*Ananas* species	N94-92 Long Fruit #2 NGRL 33
HANA176	*A. comosus* var. *comosus*	N00-10 Dole Cayenne
HANA177	*A. comosus* var. *comosus*	N94-43 Singapore
HANA178	*A. comosus* var. *comosus*	Chimpaka
HANA179	*A. comosus* var. *comosus*	3621 N03-23
HANA180	*A. comosus* var. *comosus*	153 N03-24
HANA 185	*A. comosus* var. *comosus*	N04-8

© 2013 Nanjing Agricultural University
